# Influence of Hydrogen Plasma on the Surface Structure of Beryllium

**DOI:** 10.3390/ma15186340

**Published:** 2022-09-13

**Authors:** Mazhyn Skakov, Erlan Batyrbekov, Igor Sokolov, Arman Miniyazov, Timur Tulenbergenov, Yerzhan Sapataev, Nurkhat Orazgaliyev, Olga Bukina, Gainiya Zhanbolatova, Yernat Kozhakhmetov

**Affiliations:** 1 National Nuclear Center of the Republic of Kazakhstan, Kurchatov 071100, Kazakhstan; 2 Institute of Atomic Energy Branch of National Nuclear Center of the Republic of Kazakhstan, Kurchatov 071100, Kazakhstan; 3 The Department of Applied Physics and Heat Power Engineering, Shakarim University, Semey 071412, Kazakhstan

**Keywords:** ITER, beryllium, plasma, hydrogen, erosion

## Abstract

This paper presents the research results of hydrogen plasma effect on the surface structure of the TGP-56 beryllium. In the linear simulator, the operating conditions of the first wall of ITER are simulated. Beryllium was irradiated with hydrogen plasma at surface temperatures of ~360 °C, ~800 °C, and ~1200 °C, depending on its location in the ITER chamber; with a different number of pulses with a duration of each pulse of 500 s. Samples of irradiated beryllium were subjected to a set of material studies. Experimental data were obtained on the change in the structure of the surface and edges of the beryllium samples after the plasma effect. It was found that at normal (2 MW/m^2^) and increased (4.7 MW/m^2^) heat fluxes on the first wall of the ITER, the edges and beryllium surface have good resistance to erosion. Under critical conditions close to the melting point, beryllium strongly erodes and evaporates. It has been established that this material has a high resource resistance to hydrogen plasma effect in the ITER under operating conditions.

## 1. Introduction

As it is known, to date, beryllium is a material for the first wall (FW) in the main ITER chamber facing the plasma [1]. The main characteristics substantiating this choice are the high thermal conductivity of beryllium, its compatibility with plasma, and the ability to absorb oxygen. However, there are also disadvantages when using beryllium; these include high erosion, low thermal fatigue resistance, retention of hydrogen isotopes (H, D, T), etc. [2,3,4,5,6,7]. Thermal fatigue/crack resistance are key elements since cracking can lead to higher erosion and lower thermal conductivity [2,4]. The study of the beryllium erosion by hydrogen isotopes (H, D, T) during the interaction between plasma and FW is a subject of numerous research works [4,5,6,7] and corresponding references in these papers.

The initial thickness of a beryllium tile is 8–10 mm [1,2]. Evaluation of a tile thickness loss due to erosion under normal and critical operating conditions of the ITER is an important task. As is known, the beryllium erosion refers to such physical processes as physical sputtering, physical sputtering with a chemical effect, sublimation, evaporation and melting accompanied by splashing [6,7,8,9,10]. These physical processes depend on the conditions of beryllium irradiation. The presence of an oxide layer also affects the erosion of beryllium [7,11]. The oxide layer leads to the reduction of beryllium erosion and retention of hydrogen isotopes. However, at high temperatures (T > 650 °C), it is assumed that beryllium will still diffuse through beryllium oxide and the sputtering rate will be changed by a factor of 2–3 [12,13]. Another factor affecting erosion is a beryllium surface roughness [7,14,15,16]. Erosion of the surface of rough samples is significantly reduced due to the redeposition of sputtered atoms.

The purpose of this research is to study how hydrogen plasma affect the surface and edges of beryllium plates, taking into account the thermal loads during ITER operation; and to evaluate their erosion resistance.

## 2. Materials and Methods

The research object is TGP-56 beryllium (Ø10), manufactured according to the standard of the Ulba Metallurgical Plant, JSC [17]. Workpieces were cut from the rod in the form of pellets 5.1 mm thick with a diametrical slot 1 mm wide and 2 mm deep using the electroerosive method.

**Figure 1:**
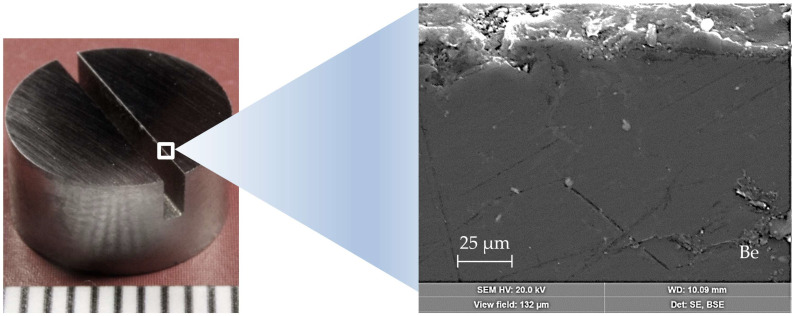
Initial beryllium sample (**left**) and an SEM image of the slit edge (**right**).

The operating conditions of beryllium panels in the ITER were implemented in a linear simulator using a plasma–beam discharge. Based on the simulation [18], the temperatures of the surface and edges of beryllium plates were determined under normal (~360 °C), increased (~800 °C), and critical (~1200 °C) thermal loads in the ITER. The impact power of the plasma–beam discharge varied depending on the calculated temperature of the surface edges of the beryllium plates. Beryllium samples were installed in the target unit of an experimental plasma–beam installation (PBI) [19,20]. The general view of the initial sample is shown in Figure 1.

The experimental unit is a linear simulator. The PBI makes it possible to test materials with an electron beam and a plasma–beam discharge, which are in pulsed and stationary modes. An electron beam is formed on the axis of the installation using an electron source, which is compressed by an electromagnetic system with an induction on the axis of 0.1 T. Further, the working gas is supplied to the vacuum chamber to a pressure of 1 × 10^−3^ Torr; a plasma–beam discharge is formed, which is transported to the interaction chamber. The energy of the ions is determined by the negative potential of the target up to minus 2000 V.

During plasma effect experiments, the temperature on the irradiated beryllium surface was controlled using a METIS M322 two-color pyrometer. From the backside, the temperature was measured with a WR-5/20 tungsten–rhenium thermocouple. During the irradiation of beryllium with hydrogen plasma, the beam–plasma discharge was monitored and diagnosed using an HR 2000+ ES optical spectrometer, a Langmuir probe, and a CIS-100 mass spectrometer. The probe measurements were processed using QtiPlot software for the analysis and visualization of data.

The roughness parameters were determined using a Mitutoyo Surftest SJ-410 profilometer on the working surface along the diametrical groove. The mass of the samples was measured using an analytical balance MS205DU with a discreteness of 0.01 mg. X-ray diffraction patterns of the samples were taken using an Empyrean diffractometer in the PIXcel1D detector mode (scanning linear detector). The diffraction patterns were processed using the HighScore program. The phase composition was identified using the Crystallography Open Database (hereinafter COD) [21] and the PDF-2 ICDD Release 2004 database. A visual inspection was carried out in order to establish the nature of the damages of the tested samples according to macrophotographs of their appearance. The surface morphology was studied on a scanning electron microscope Tescan Vega3. The microstructure of the samples was studied using a SopTop ICX-41M optical microscope in polarized light mode at magnifications up to ×500. The cutting of the samples for studying the microstructure was performed perpendicular to the groove and diametrically to the focus of plasma effect; the wire-cut method was used in an ARTA 123 PRO electroerosion machine. Fine grinding of the samples surface was carried out using a DualPrep 3 grinding and polishing machine with water cooling. When preparing the surface, SiC sanding paper with a grain size of up to P2500 and a diamond suspension with a grain size of 6.00–0.25 µm were used.

## 3. Results and Discussion

To ensure the effect of hydrogen ions on beryllium from a beam–plasma discharge, an electric potential of minus 2000 V was applied to the target. The speed of temperature increase to the calculated value was ~100 °C/s. When the sample was exposed to plasma, the time shelf of each impulse was 500 s, according to the impulse time in the ITER [22]. A pause was provided between impulses to cool the sample to a temperature of 150 °C. The main parameters of the experiments are given in Table 1.

There were no direct measurements of the ion temperature in these experiments due to the complexity of the correct application of this method at the present moment in the facility. Data registration was carried out in real time during experiments on the interaction of hydrogen plasma with beryllium in all the irradiation modes.

**Figure 2:**
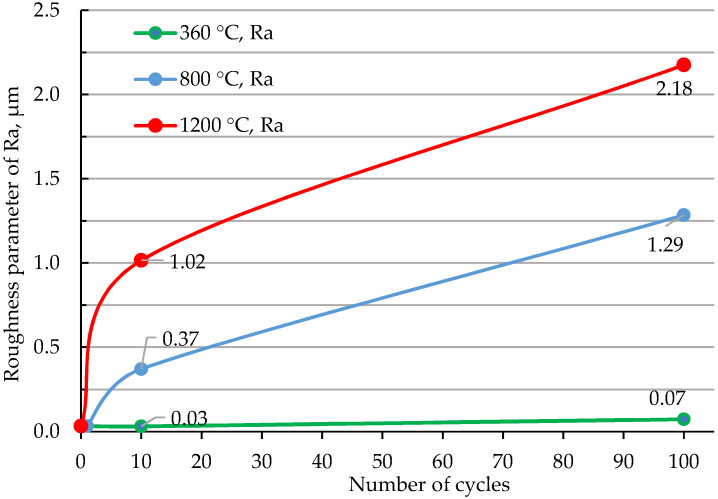
Dependence of the surface roughness parameters of the beryllium samples on the conditions of effect to hydrogen plasma.

The parameter “Ra” of the roughness of the samples was determined on the working surface along the diametrical slot. The arithmetic mean of the roughness profile (Ra) of the original samples was 0.032 ± 0.004. The results of measuring the roughness parameters depending on the irradiation temperature and number of impulses are shown in Figure 2.

It can be seen from Figure 2 that Ra increases with the growth of plasma effect temperature and the cycle numbers. Analyzing the above dependence, it can be observed that it is the erosion of the beryllium surface that occurs; this is because beryllium is actively evaporated at a temperature of about 730 °C. In this case, the change in roughness begins at a temperature of 360 °C. Based on the graph data (Figure 2), it can be seen that erosion has a direct dependence on temperature, as well as on the number of test cycles.

**Figure 3:**
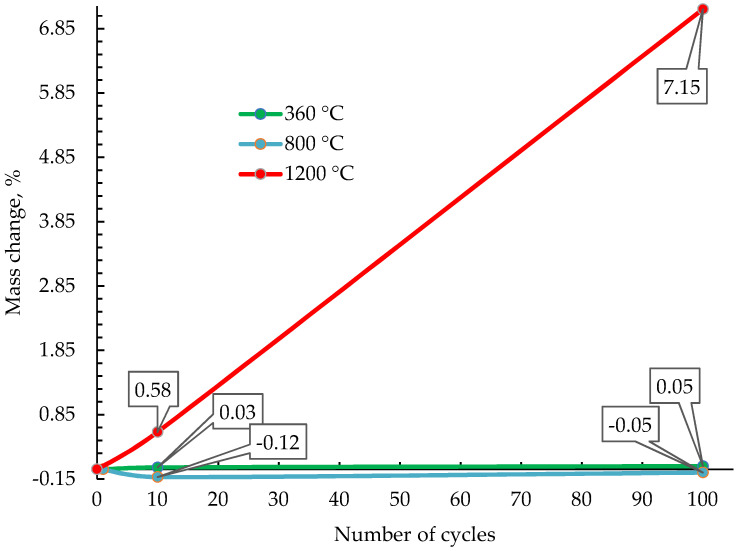
Dependence of the surface roughness parameters of the beryllium samples on the conditions of exposure to hydrogen plasma.

Figure 3 shows a dependency graph of the change in the beryllium mass from temperature and the number of impulses based on the results of 10 weighings.

It can be seen from the figure that the largest mass changes (to 7%) are observed for the Be-3 and Be-6 samples tested at a temperature of 1200 °C and with the number of cycles 10 and 100, respectively. Here, it can be seen that as the ion fluence (Table 1) increases by 10 times; the mass loss also changes by 10 times between samples Be-3 and Be-6. In the other test modes, the mass change of the samples is insignificant.

**Figure 4:**
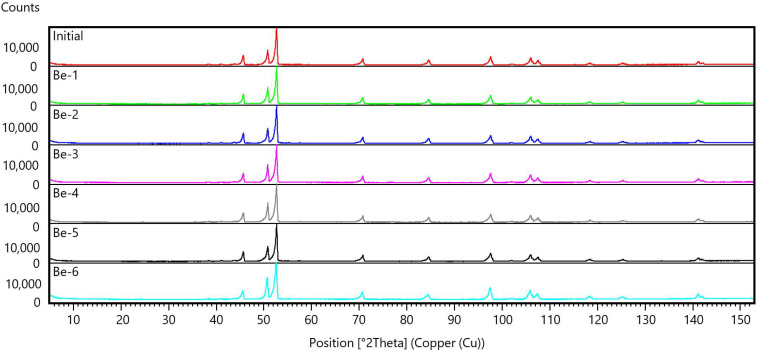
General view of the X-ray diffractograms of Be.

Figure 4 shows an X-ray diffraction pattern of the beryllium surface. The basis of the phase composition is the metallic beryllium phase. The beryllium peaks in the diffractograms are smeared towards smaller diffraction angles. This is a consequence of the high transparency of beryllium for X-ray radiation. Peaks of low intensity in the angle range of 37–44° 2θ are identified as belonging to the beryllium oxide phase.

The results of a quantitative assessment of the content of the detected phases by the corundum number method are presented in Table 2.

The presence of BeO is due to the presence of oxygen and an oxide film in the composition of the beryllium grades TGP-56. The content of the oxide phase in all the Be samples is at the level of 3 wt.% and after exposure to hydrogen plasma does not change. 

Changes in beryllium phase composition are not observed after exposure to hydrogen plasma.

As a result of microstructural studies, areas were selected on the surfaces and transverse sections of the beryllium samples; located in the center of the plasma flow with the parameters shown in Table 1.

### 3.1. Be-1 and Be-4 Samples (360 °C)

**Figure 5:**
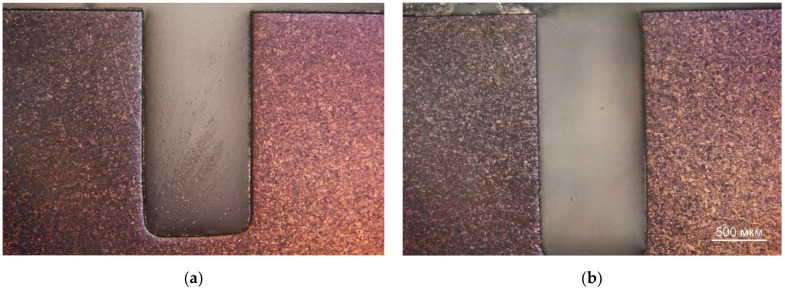
Microstructure of the samples cross section (360 °C): (**a**) Be-1 (10 impulses); and (**b**) Be-4 (100 impulses).

In the samples of the first group, no obvious changes in the surface and microstructure were found (see Figure 5).

**Figure 6:**
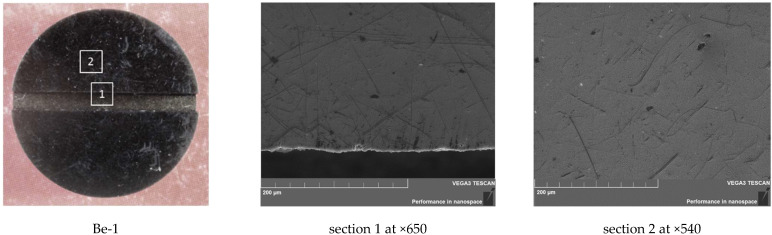
SEM images of the sample Be-1 surface (360 °C, 10 cycles): (**a**) Be-1; (**b**) section 1 at ×650; and (**c**) section 2 at ×540.

Scanning electron microscopy images of the sample Be-1, shown in Figure 6, also confirm the absence of changes on the working surface.

**Figure 7:**
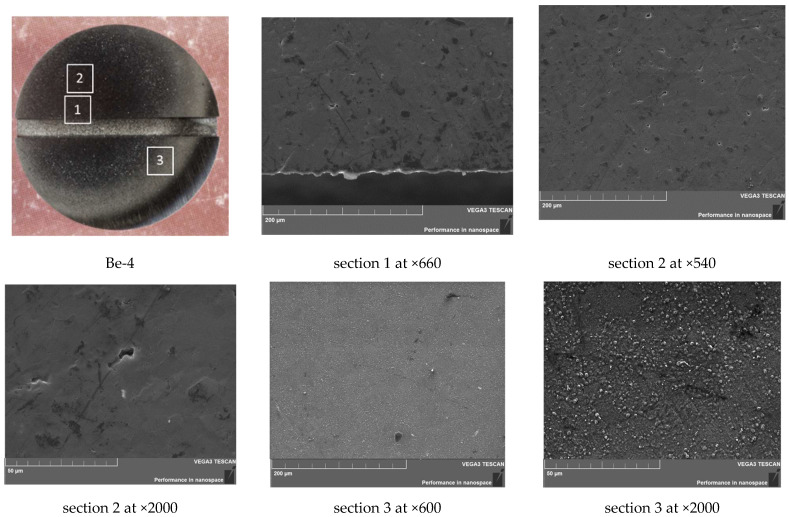
SEM images of the sample Be-4 surface (360 °C, 100 cycles).

An increase in the number of cycles to 100 at a temperature of 360 °C leads to the appearance of signs of erosion. The selected areas for sample Be-4 have varying degrees of erosion as they move away from the center of the sample. In sections 1 and 2 (see Figure 7), in addition to traces of surface etching, the presence of pores 5–15 µm in size with low dispersion is observed. In section 3, there are almost no pores and there is a dense accumulation of rounded bubbles with a diameter of about 2–3 μm; these were formed during the blistering process (Figure 7, section 3). The surface of sample Be-4 has a matte finish with temper colors along the edges. Obviously, this is due to a change in the structure of the oxide film; this is present on the original beryllium (see Table 2) and retains its structure after 10 impulses.

### 3.2. Be-2 and Be-5 Samples

This group of samples tested under a temperature of 800 °C exhibits large-scale erosion of the working surface.

**Figure 8:**
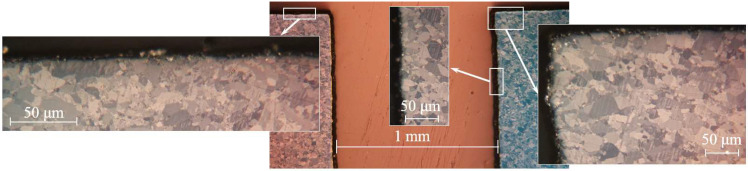
Microstructure of the Be-4 sample cross section (360 °C, 100 cycles).

The microstructure of sample Be-2 is shown in Figure 8. Studies of thin sections of sample Be-2 showed that a load with a temperature of 800 °C and 10 cycles causes relatively shallow erosion; in addition, the walls and bottom of the cut remain intact. The pore depth reaches to 5~μm.

**Figure 9:**
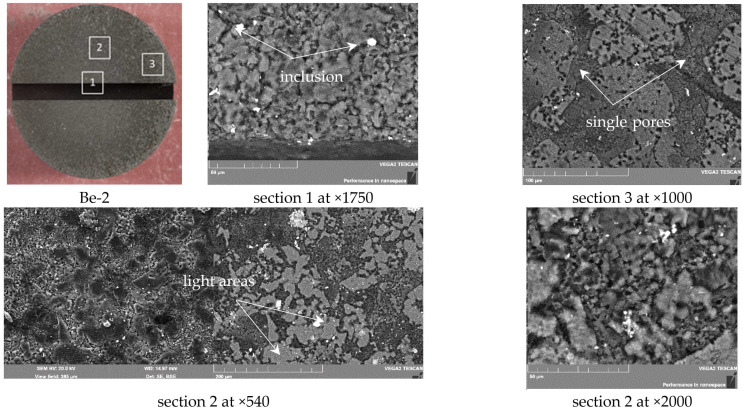
SEM images of the surface of sample Be-2 (800 °C, 10 cycles).

Figure 9 shows that the entire surface of sample Be-2 was eroded. In section 1, it is possible to notice a uniform porous structure; here, the matrix represents rounded particles (5–10 µm in size) and pores in the form of veinlets up to 5 µm wide. In the body of the matrix, there are beryllium oxide inclusions up to 5 µm in size; these are predominantly round in shape, which is confirmed by the results of the X-ray analysis.

With distance from the center of the sample, the pore sizes decrease and larger particles predominate in the matrix; moreover, areas (up to 100 μm in size) with a light shade are additionally formed (Figure 9, section 2). Here the proportion of light areas is about 50%. Their surface is covered with a finely porous structure of submicron size (Figure 9, section 2 at ×2000). Such a change in the surface occurs due to high thermal stresses and intense erosion. The surface layer of beryllium samples is subjected to melting, evaporation, and re-solidification [23].

In section 3 (see Figure 9), the formation of a porous structure is invisible. The share of coverage of light areas reaches 65%, and its sizes are up to 150 µm. Single rounded pores of about 5 µm in diameter are visible on the surface of light areas. The surface Be-2 sample has a uniform dark gray shade with no visible signs of form change.

**Figure 10:**
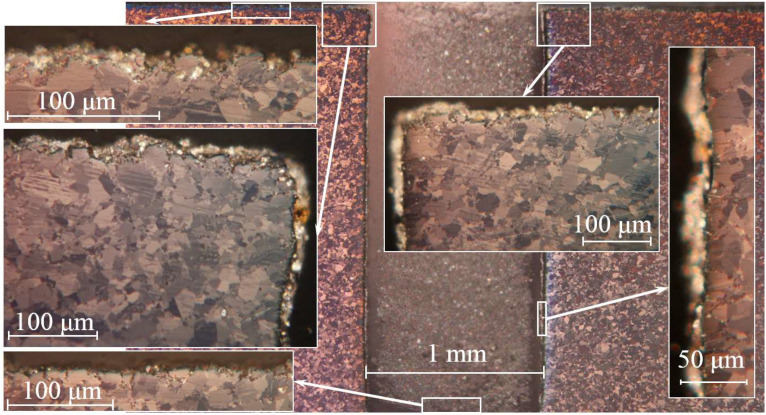
Microstructure of the Be-5 sample cross section (800 °C, 100 cycles).

An increase in the number of test cycles led to the erosion of sample Be-5 not only of the near-surface layer with the formation of pores of up to 15 µm deep (see Figure 10), but also to the erosion of the walls and bottom of the notch. The walls remain relatively even and smooth, while pores of up to 8 µm deep form on the bottom surface.

**Figure 11:**
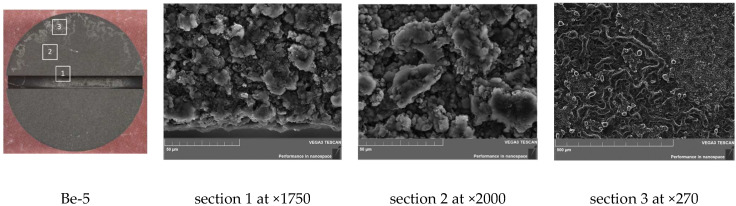
SEM images of the Be-5 sample surface (800 °C, 100 cycles).

Almost the entire surface of sample Be-5 is eroded and has a uniform, porous structure similar to Section 1 of sample Be-2; however, it has a complex morphology similar to a fractal-like structure (Figure 11). The surface of Be-5 has a dark gray tint. Along the periphery of the upper segment of the working surface of the sample, there are curly-shaped lines of a light gray shade.

### 3.3. Be-3 and Be-6 Samples

The analysis shows that the surface morphology of the samples of this group differs significantly from the other samples. The surface of the samples has deep pores (channels) with a finely porous coating.

**Figure 12:**
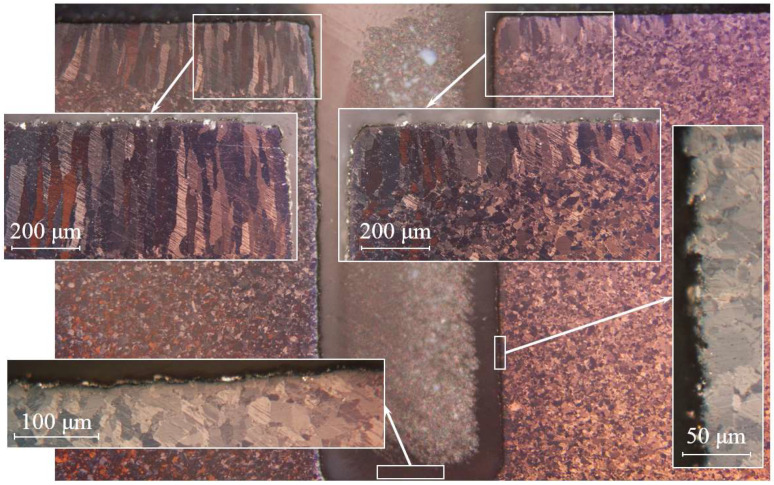
Microstructure of the Be-3 sample cross section (800 °C, 100 cycles).

On the surface area of sample Be-3, a recrystallization area with a typical oriented (columnar) grain structure is observed up to a depth of ~350 µm from the surface (Figure 12). The recrystallization zone may have formed during the α→β phase transition. The geometry of the cut and the surface did not change. The pore depth on the surface reaches up to 30 µm.

**Figure 13:**
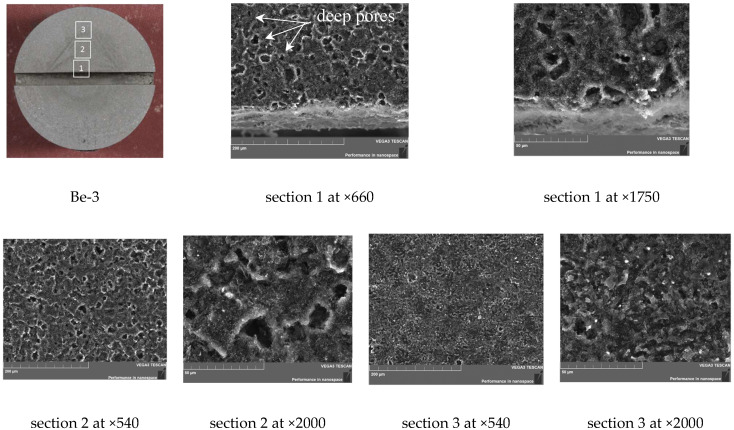
SEM image of the sample Be-3 surface (1200 °C, 10 cycles).

As can be seen in Figure 13, the concentration of pores in sections 1 and 2 of sample Be-3 is the same; and the average value of the pore diameter in these sections is about 20 µm. In the area remote from the center of the sample (Figure 13, section 3), the concentration and the average value of the pore diameter decrease by 10 µm. The surface of the matrix is covered with a fine structure. The working surface has a light gray shade and a slight change in shape in the central part.

**Figure 14:**
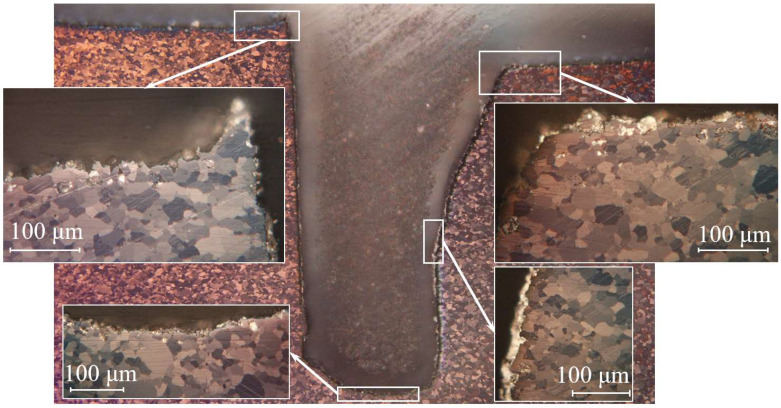
Microstructure of the Be-6 sample cross section (1200 °C, 100 cycles).

Sample Be-6 was subjected to change in shape not only due to plasma effect, but also to deformation during the test. Under plasma exposure, sample erosion occurred on the entire surface of the sample; this included the walls and bottom of the notch (Figure 14). The surface of the right segment is more eroded than the left one.

Moreover, in a certain area of the notch, a decrease in the width of the notch from 1.00 mm to 0.77 mm was found. This effect is due to the reprecipitation of beryllium particles eroded from the surface; since at a beryllium temperature above 1000 K, there is a significant increase in erosion due to thermal evaporation.

**Figure 15:**
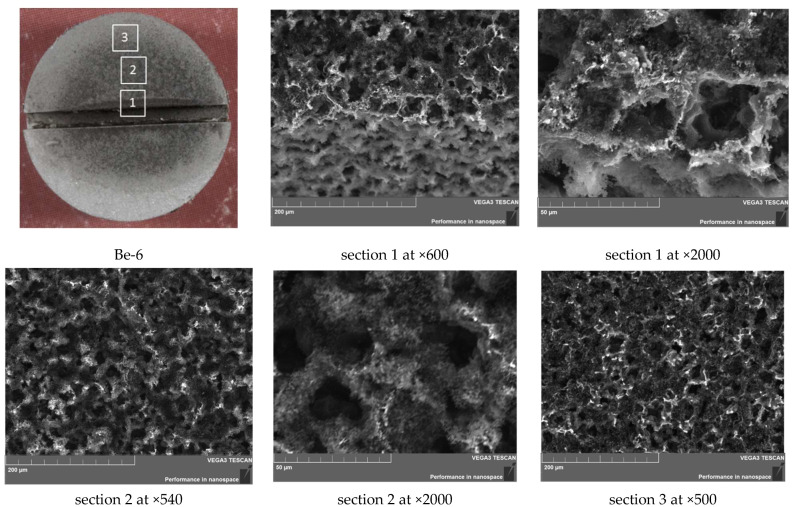
SEM images of the Be-3 sample surface (1200 °C, 100 cycles).

The surface of sample Be-6 is similar to that of sample Be-3: deep pores (channels) are also present; however, in this case, their concentration is higher and the pore diameter reaches 30 µm (see Figure 15). On the surface, there is a clear change in shape in the form of a depression and darkening in the central part of the sample.

The highest temperature and the most aggressive region of plasma–beam discharge action is located near the slot; this is because there is no heat removal through the side walls of the samples to the holder, and the plasma is maximally concentrated. As can be seen from the SEM images, off-center areas 2 and 3 are similar and less damaging than in area 1.

## 4. Conclusions

Thus, in this research, experimental material studies were carried out concerning the impact of hydrogen plasma on TGP-56 type beryllium; as a candidate material for the first wall in the main ITER chamber facing the plasma. During irradiation, the number of pulses changed from 10 to 100 with the same duration of 500 s under the surface temperatures of ~360 °C, ~800 °C, and ~1200 °C.

Experimental data are obtained on the change in the surface morphology of beryllium as a result of irradiation. It is established that the change in roughness has an almost direct linear dependence, both on the increase in temperature and on the number of cycles.

There is almost no change in the mass of samples irradiated at temperatures of ~360 °C and ~800 °C; this positively characterizes the test material. The main mass change occurs in samples irradiated at ~1200 °C, which is associated with large-scale surface erosion and material evaporation.

The basis of the phase composition of the sample in the initial state is the hexagonal phase of metallic beryllium. The peaks of beryllium in the diffraction patterns are getting a bit unclear near the smaller angles, which is associated with the transparency of beryllium for X-ray radiation. The ratio of the beryllium phase to the beryllium oxide phase remains constant.

According to the results of the microstructural analysis, it was established that under the action of hydrogen ions in the near-surface layers of beryllium at temperatures above 800 °C, a porous structure of micron and submicron sizes is formed. An increase in the number of cycles from 10 to 100 leads to the development of the linear dimensions of the porous structure. Significant erosion of the surface of beryllium occurs at a temperature of 1200 °C. Moreover, an increase in the number of impulses leads to an increase in the number and size of the pores induced by irradiation.

It is established that at a test temperature of ~1200 °C, recrystallization of the surface layer is possible due to the formation of a characteristic oriented structure upon cooling. Apparently, beryllium with a surface-oriented structure can have a significant resistance to erosion under ionic and thermal impact.

It is found that the influence of hydrogen plasma at “normal” and “increased” heat flux in the ITER will not have a critical effect on the destruction of the edges and surfaces of panels made of the beryllium grades TGP-56 (Be ≥ 96.8%); this is so even after 100 operating impulses. Thus, this material has a high resource resistance to the impact of hydrogen plasma in the ITER under operating conditions. It is shown that at a surface temperature of ~1200 °C, destruction can be critical for a given grade of beryllium; plasma contamination in the ITER chamber will occur, which will lead to the premature replacement of beryllium panels.

It is known that the reference grades of beryllium for ITER (S-65, CN-G01, TGP-56FW) are characterized by a Be > 99% purity. In this work, less “pure” grade beryllium was tested, which is below the ITER and nuclear standards. Nevertheless, the practical significance of the results obtained is of great importance for a more detailed understanding of the structural changes in beryllium FWP during operation under ITER conditions.

At the moment, we continue to study changes in the structure of beryllium plates under ITER operating conditions. In particular, measurements of the thermal conductivity of beryllium samples and TEM studies will be carried out to study the defect structure after plasma exposure. It is also planned to test titanium beryllides under ITER operating conditions.

## Figures and Tables

**Table 1:** Parameters for conducting experiments on the effect of hydrogen plasma on the beryllium samples.

# Sample	Temperature of Irradiated Surface, °C	Ion Current Density, mA/cm^2^	Plasma Concentration, cm^−3^	Electronic Temperature, eV	Ionic Flow, m^−2^s^−1^	Fluence of Ions, m^−2^	Number of Impulse
Be-1	360 ± 20	~179	3.09 × 10^17^	6.02	1.47 × 10^21^	7.34 × 10^24^	10
Be-2	800 ± 20	~208	1.58 × 10^17^	13.36	1.1 × 10^21^	5.53 × 10^24^	10
Be-3	1200 ± 20	~173	1.9 × 10^18^	3.9	7.12 × 10^21^	3.56 × 10^25^	10
Be-4	360 ± 10	~185	3.09 × 10^17^	6.02	1.47 × 10^21^	7.34 × 10^25^	100
Be-5	800 ± 10	~173	1.58 × 10^17^	13.36	1.1 × 10^21^	5.53 × 10^25^	100
Be-6	1200 ± 10	~180	1.9 × 10^18^	3.9	7.12 × 10^21^	3.56 × 10^26^	100

**Table 2:** The results of the evaluation of the quantitative content by the method of corundum numbers in the automatic mode of the “HighScore” program.

	Be-1	Be-2	Be-3	Be-4	Be-5	Be-6	Be (Initial)
Be (P63/mmc)	96.6	96.8	96.7	96.7	96.8	96.6	96.6
BeO (P63mc)	3.4	3.2	3.3	3.3	3.2	3.4	3.4

## Data Availability

Not applicable.

## References

[1] Barabash V., Akiba M., Mazul I., Ulrickson M., Vieider G. (1996). Selection, development and characterisation of plasma facing materials for ITER. J. Nucl. Mater..

[2] Conn R.W., Doerner R.P., Won J. (1997). Beryllium as the plasma-facing material in fusion energy systems—Experiments, evaluation, and comparison with alternative materials. Fusion Eng. Des..

[3] Mitteau R., Eaton R., Gervash A., Kuznetcov V., Davydov V., Rulev R. (2017). Allowable heat load on the edge of the ITER first wall panel beryllium flat tiles. Nucl. Mater. Energy.

[4] Spilker B., Linke J., Loewenhoff T., Pintsuk G., Wirtz M. (2019). Performance estimation of beryllium under ITER relevant transient thermal loads. Nucl. Mater. Energy.

[5] Kozhakhmetov Y.A., Skakov K., Kurbanbekov S.R., Mukhamedov N.M. (2021). Powder Composition Structurization of the Ti-25Al-25Nb (at.%) System upon Mechanical Activation and Subsequent Spark Plasma Sintering. Eurasian Chem. J..

[6] Kupriyanov I., Nikolaev G., Kurbatova L., Porezanov N., Podkovyrov V., Muzichenko A., Zhitlukhin A., Gervash A., Safronov V. (2015). Erosion of beryllium under ITER—Relevant transient plasma loads. J. Nucl. Mater..

[7] De Temmerman G., Heinola K., Borodin D., Brezinsek S., Doerner R.P., Rubel M., Fortuna-Zaleśna E., Linsmeier C., Nishijima D., Nordlund K. (2021). Data on erosion and hydrogen fuel retention in Beryllium plasma-facing materials. Nucl. Mater. Energy.

[8] Brezinsek S., Stamp M.F., Nishijima D., Borodin D., Devaux S., Krieger K., Marsen S., O’Mullane M., Björkas C., Kirschner A. (2014). JET EFDA Contributors. Nucl. Fusion.

[9] Nordlund K., Björkas C., Vörtler K., Meinander A., Lasa A., Mehine M., Krasheninnikov A.V. (2011). Mechanism of swift chemical sputtering: Comparison of Be/C/W dimer bond breaking. Nucl. Instrum. Methods Phys. Res. B.

[10] Jepu I., Matthews G., Widdowson A., Rubel M., Fortuna-Zalesna E., Zdunek J., Petersson P., Thompson V.K., Dinca P., Porosnicu C. (2019). Beryllium melting and erosion on the upper dump plates in JET during three ITER-like wall campaigns. Nucl. Fusion.

[11] Doerner R., Björkas C., Nishijima D., Schwarz-Selinger T. (2013). Erosion of beryllium under high-flux plasma impact. J. Nucl. Mater..

[12] Roth J.W.R., Wampler W., Jacob J. (1997). Mechanism of swift chemical sputtering: Comparison of Ce/C/W dimer bond breaking. Nucl. Mater..

[13] Doerner R.P. (2005). Beryllium and Liquid Metals as Plasma Facing Materials. Nucl. Fusion Res..

[14] Duensing F., Hechenberger F., Ballauf L., Reider A.M., Menzel A., Zappa F., Dittmar T., Böhme D.K., Scheier P. (2021). Energetic D+ and He+ impinging on solid beryllium: Observation of physical and chemically assisted atomic and molecular ion sputtering. Nucl. Mater. Energy.

[15] Küstner M., Eckstein W., Hechtl E., Roth J. (1999). Angular dependence of the sputtering yield of rough beryllium surfaces. J. Nucl. Mater..

[16] Szabo P., Cupak C., Biber H., Jäggi N., Galli A., Wurz P., Aumayr F. (2022). Analytical model for the sputtering of rough surfaces. Surf. Interfaces.

[17] Beryllium Blanks [Electronic Source]—URL. http://www.ulba.kz/ru/production2_06.htm.

[18] Sokolov I.A., Skakov M.K., Miniyazov A.Z., Aubakirov B.T., Tulenbergenov T.R., Gradoboev A.V. (2021). Analysis of the beryllium stability under standard and critical operation in a fusion reactor. Eurasian J. Phys. Funct. Mater..

[19] Sokolov I.A., Skakov M.K., Zuev V.A., Ganovichev D.A., Tulenbergenov T.R., Miniyazov A.Z. (2017). Study of the Interaction of Plasma with Beryllium That Is a Candidate Material for the First Wall of a Fusion Reactor. Tech. Phys..

[20] A Sokolov’ I., Skakov M.K., Miniyazov A.Z., Tulenbergenov T.R., Zhanbolatova G.K. (2021). Interaction of plasma with beryllium. J. Physics: Conf. Ser..

[21] Gražulis S., Chateigner D., Downs R.T., Yokochi A.F.T., Quirós M., Lutterotti L., Manakova E., Butkus J., Moeck P., Le Bail A. (2009). Crystallography Open Database—An open-access collection of crystal structures. J. Appl. Cryst..

[22] Anand H., Pitts R., De Vries P., Snipes J., Nespoli F., Labit B., Galperti C., Coda S., Brank M., Kos L. (2019). Experimental implementation of a real-time power flux estimator for the ITER first wall on the TCV tokamak. Fusion Eng. Des..

[23] Kupriyanov I., Porezanov N., Nikolaev G. (2014). Study of beryllium damage under iter-relevant transient plasma and radiative loads. Fusion Sci. Technol..

